# IGF and IGFBP as an index for discrimination between vitamin D supplementation responders and nonresponders in overweight Saudi subjects

**DOI:** 10.1097/MD.0000000000010702

**Published:** 2018-05-11

**Authors:** Nasser M. Al-Daghri, Sobhy M. Yakout, Kaiser Wani, Malak Nawaz Khan Khattak, Spiro D. Garbis, George P. Chrousos, Omar S. Al-Attas, Majed S. Alokail

**Affiliations:** a Prince Mutaib Chair for Biomarkers of Osteoporosis, Biochemistry Department, College of Science, King Saud University, Riyadh, Saudi Arabia; b Centre for Proteomic Research, Institute for Life Sciences, University of Southampton, Southampton, UK; c First Department of Pediatrics, University of Athens, Athens, Greece.

**Keywords:** growth hormone, IGF-I, IGF-1/IGFBP-3 ratio, vitamin D

## Abstract

Vitamin D deficiency is common in the Kingdom of Saudi Arabia (KSA). Therefore, it is significant to recognize which biochemical markers modulate serum 25 hydroxyvitamin D (25(OH)D) in response to vitamin D supplementation in such a population. Our aim was to study the correlation of insulin-like growth factor (IGF) and insulin growth factor binding protein (IGFBP) with serum 25(OH)D in response to vitamin D supplementation in a Saudi population. A total of 199 (89 males/110 females) vitamin D deficient subjects (25(OH)D level <50 nmol/L), aged 40.4 ± 11.4 years, were given vitamin D supplements (50,000 IU/mL every week) for the first 2 months, then twice a month for 2 months, followed by daily 1000 IU in the last 2 months. Fasting blood samples were taken at baseline and 6 months after the final dose of vitamin D. Serum 25(OH)D, IGF-1 and IGF-2, and IGFBPs 2–5 were measured. Vitamin D response was computed for all subjects as the difference in levels of serum 25(OH)D concentration at the end of 6 months compared to baseline. After intervention, serum 25(OH)D concentration significantly increased from 35.6 nmol/L (26.6–43.5) to 61.8 nmol/L (54.8–73.3) in responder subjects (*P* < .01) and from 35.1 nmol/L (21.2–58.2) to 38.3 nmol/L (25.5–48.3) in nonresponders (*P* = .13). Subjects with lower baseline serum IGF-II, IGFBP-2, and IGF-1/IGFBP-3 ratio are more sensitive to acute vitamin D status changes. IGF1 and IGF-1/IGFBP-3 ratio significantly increased in all subjects after 6 months (*P* = .01). Changes in 25(OH)D was significantly associated with changes in IGFBP-2 and IGF-1/IGFBP-3 ratio in responders only. This study proposes that changes in circulating IGF-I and IGFBP-3 are modulated by vitamin D supplementation and can be taken into consideration in investigations involving vitamin D correction. Moreover, increase in serum 25(OH)D and IGF-I/IGFBP-3 molar ratio are more sensitive markers for the response to vitamin D supplementation in Saudi population.

## 1 Introduction

Recently, insulin-like growth factors (IGFs) have gained interest among scientists in knowing how the IGF system disruption is related to metabolic disease like growth deficiency, obesity, cancer, neurological, and cardiovascular diseases and also how its components can used as biomarkers of disease and/or a targeted for their treatment.^[1]^ IGFs include IGF-1 and IGF-2, polypeptide hormone highly homologous to insulin that synthesized by liver and some other organs under the influence of somatotropin (growth hormone).^[2]^ They play a significant part in growth, differentiation, and cellular metabolism.^[3]^ Additionally, the IGF system contains 6 IGF binding proteins (insulin growth factor binding protein [IGFBPs] 1–6) and are major regulators of the IGF activity.^[4]^ In the circulation, majority of IGF molecules bind to IGFBPs leaving a small fraction free that can bind to its receptor and begin signaling survival and cell proliferation.^[5]^ The IGFBPs bound to IGFs increase their half-life and alter their function or facilitate their passage to the target tissues.^[6]^ Free IGF-I is increased in overweight and obese patients with negative correlation with IGFBP-1, IGFBP-3.^[7]^ Low IGF-I and high IGFBP-3 are linked with increased waist-to-hip ratio.^[8]^

Furthermore, serum 25 hydroxyvitamin D (25(OH)D) was found to be linked to metabolic syndrome (MetS), with the IGF system interaction recently highlighted.^[9]^ Previous studies have shown positive associations between 25(OH)D and IGF-1 concentrations in healthy adults.^[10]^ IGF-1 increases 1,25(OH)D level in vitro by stimulating 1α-hydroxylase expression.^[11]^ In healthy men, IGF-1 treatment increases free vitamin D index.^[12]^ On the other hand, other studies propose that vitamin D status can be used as a good indicator of IGF-1 concentrations; and a better vitamin D status may stimulate to get normal IGF-1 values.^[13]^ Also, a significant rise in serum IGF-1 was in response to vitamin D in nutritional rickets children.^[14]^

Given the strong relationship between IGF and vitamin D as well as stimulation of IGF and IGFBP by 1,25(OH)D^[15]^ in fat tissues, we suggested that supplementation of vitamin D may modulate parameters of the IGF system. To test this hypothesis, we prospectively measured these biomarkers in the sera of overweight subjects who participated in a 6-month interventional study with high-dose vitamin D supplementation.

## 2 Material and methods

### 2.1 Study population

This 6-month prospective study was part of the Vitamin D Interventional Trial series of the Prince Mutaib Chair for Biomarkers of Osteoporosis (PMCO), King Saud University (KSU), Riyadh, Kingdom of Saudi Arabia (KSA). Ethical approval was obtained from the ethics committee of the College of Medicine Research Center, KSU in Riyadh, KSA. A total of 199 (89 males/110 females) vitamin D deficient subjects [25(OH)D <50 nmol/L), aged 40.4 ± 11.4 years, were admitted in the study. Anyone with anemia, type 2 diabetes mellitus, cancer, cardiovascular disease, liver and renal dysfunction, or thyroid dysfunction has been excluded from the study. Subjects taking vitamin D supplements in the last 6 months before intervention was excluded at the screening phase.

### 2.2 Anthropometry and blood collection

All the individuals were asked to visit primary heath care centers for blood sampling and anthropometrics including weight, height, waist, and hip circumference, and mean diastolic and systolic blood pressure were measured on an assigned date. Body mass index (BMI) was calculated by dividing weight (kilograms) by height (square meters) (kg/m^2^). About 5 mL of fasting venous blood samples were collected from each individual and processed for separation of serum samples. The remaining blood and serum samples were transported to the Biomarkers Research Program in KSU, Riyadh, KSA in specialized containers for biochemical analyses and storage at −80 °C.

### 2.3 Vitamin D intervention

Oral 50,000 IU cholecalciferol (VitaD50000) (Synergy Pharma, Dubai, UAE) tablet was given weekly for first 2 months, then twice a month for next 2 months, followed by daily 1000 IU (VitaD1000) (Synergy Pharma, Dubai, UAE) for the last 2 months in all subjects. To ensure compliance, patients were asked to return unused tablets at every follow-up visit before giving another set of supplements to determine compliance. They were also regularly encouraged through Short Message Service to take vitamin D recommended dose. The orientation and intervention was conducted by qualified nutritionist, physician, and nurses in respective health care center and all the procedures followed ethical principles advised in declaration of Helsinki. The intervention study was approved by the Ethics Committee of the College of Science, KSU, Riyadh. Blood samples (5 mL) were obtained at baseline and after 6 months to monitor achievement of full vitamin D status correction. For stratification postintervention, responder was defined as those who achieved 25(OH)D above 50 nmol/L, while nonresponders were those who achieved <50 nmol/L.

### 2.4 Biochemical analyses

Fasting serum samples were analyzed for lipid profile and glucose levels in all participants using routine chemical analyzer (Konelab20XTi, Thermo Electron Corporation, Vantaa, Finland). COBAS e-411 automated analyzer (Roche Diagnostics, Indianapolis, IN) was used for measuring Serum 25(OH)D. The inter- and intraassay was applied for the estimation of serum 25(OH)D, coefficients of variation (CV) were taken 8.0% and 5.6%, respectively, with a lower detection limit (LOD) of 50 nmol/L.

### 2.5 Luminex assays for IGF-1, IGF-2, and IGFBP2–5

The Luminex kits were obtained from Millipore (Billerica, MA) and assays were conducted as per manufacturer's instructions to determine the serum levels of IGF-1, IGF-2, and IGFBP2–5 proteins. Properly diluted serum samples were incubated with the antibody-coupled microspheres and then with biotinylated detection antibody before the addition of streptavidin-phycoerythrin. The captured bead complexes were measured with FLEXMAP 3D system (Luminex Corporation, Austin, TX) using the following instrument settings (events/bead, 35; sample size, 50 μL; discriminator gate, 8000–15,000). The raw data (mean fluorescence intensity) were collected and further processed for calculating protein concentration.

### 2.6 Statistical analysis

Data were analyzed using SPSS (version 21, IBM). Continuous data were presented as mean ± standard deviation (SD) for variables following Gaussian variables and non-Gaussian variables were presented in median (1st and 3rd quartiles). All continuous variables were checked for normality using Kolmogorov–Smirnov test. Non-Gaussian variables were log transformed prior to parametric analysis. Independent *t* test and paired *t* test (pre and post) were used to check mean differences in Gaussian variables and Mann–Whitney *U* and Wilcoxon tests (pre and post) were used for non-Gaussian variables, whichever is more applicable. Multiple linear regression analysis was done as delta Δ 25(OH)D as dependent variable. Correlations between variables were done using Pearson correlation analysis. *P* value <0.05 was considered statistically significant.

## 3 Results

Table 1 describes the anthropometric and metabolic characteristics of all subjects overtime. The mean age group of the cohort was 40.4 ± 11.4 years. Baseline BMI of the subjects was 29.8 ± 4.9 and fell into the overweight category. The majority of patients were severely vitamin D deficient. Baseline serum 25(OH)D3 level was <10 nmol/L in 3% of the patients and 38% had levels ≤30 nmol/L. Over the 6 month intervention, most of the metabolic parameters, diastolic blood pressure, glucose, triglycerides, and total cholesterol, remained insignificantly different from one another with the exception of HDL-cholesterol, which showed significant improvement in 6 months than baseline (*P* = .002) and circulating levels of 25(OH)D, which significantly increased at 6 months than baseline (*P* < .001). IGF-I, IGF-II, and IGF-1/IGFBP-3 ratio at the 6-month follow-up were significantly higher than baseline (*P*-values < .001, .049 and <.001, respectively) (Table 1). IGFBP-4 was significantly lower after 6-month follow-up (*P* = .04). Vitamin D treatment increased the mean 25(OH)D level from 35.4 to 54.4nmol/l (*P* < .001). In total, 52% of the patients achieved a serum 25(OH)D level >50 nmol/L, and only 13% of patients achieved a serum 25(OH)D level >75 nmol/L.

**Table 1:**
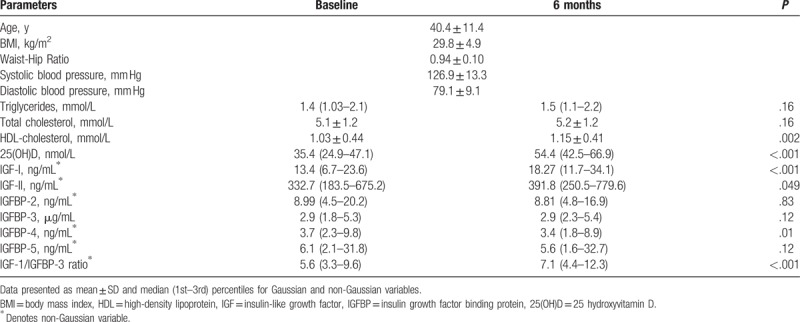
Clinical characteristics of all subjects before and after 6 mo.

The differences that were found between responders and nonresponders at baseline are shown in Table 2. There were no significant differences between responders and nonresponders to vitamin D supplementation on demographic variables like sex, age, BMI, systolic, and diastolic blood pressure at baseline. Responders had significantly higher triglycerides at baseline. At both baseline responder has significantly lower IGF-II, IGFBP-2, and IGF-1/IGFBP-3 ratio than nonresponders. Furthermore, responders have significantly high IGFBP-3 and IGFBP-5.

**Table 2:**
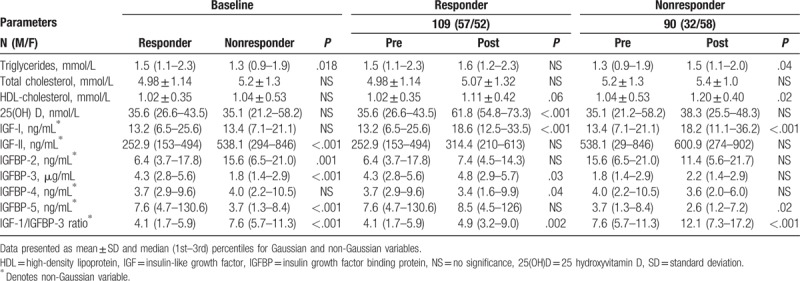
Differences at baseline and 6-mo follow-up between responders and nonresponders.

The mean responses within the each responder and no responders groups (pre and post) were also shown in Table 2. The mean 25(OH)D significantly (*P* < .001) increased by 26.1 nmol/L in responder group and by only 3.2 nmol/L in nonresponder group after postintervention. The range in serum 25(OH)D responses was 54.8 to 73.3 nmol/L. Triglycerides increased significantly (*P* = .018) in nonresponder groups while IGFBP-3 increases significantly in responder groups (*P* = .026). IGF-I and IGF-1/IGFBP-3 ratio increases significantly in both responder and nonresponder groups after postintervention (*P* < .01). IGFBP-4 decreases significantly in responder groups while IGFBP-5 decreases significantly in nonresponder groups.

**Figure 1:**
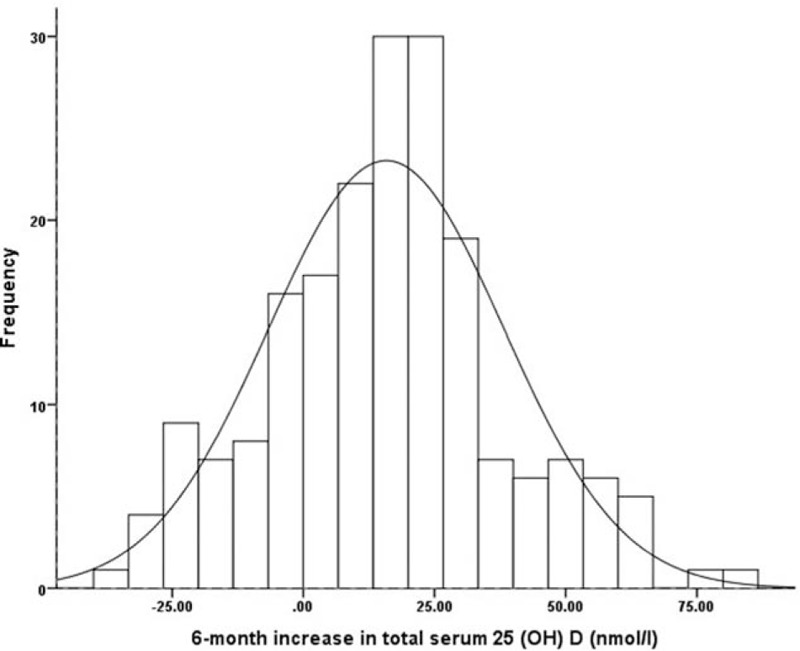
Frequency distribution of 6-month increase in serum 25(OH)D levels in 199 subjects in vitamin D supplements.

The distribution of the 6-month increase of serum 25(OH)D was shown in Fig. 1. As was visually evident, the distribution is normal (Kolmogorov–Smirnov test, *P* = .08). Of greater interest was the great spread of response variance and a wide variation of serum 25(OH)D concentration in response to trial vitamin D supplementation was observed in the 199 subjects. The mean 6-month total serum 25-OHD increase was 15.92 nmol/L, the SD was 22.42 nmol/L, the range was from −39.38 to 81.63 nmol/L, and the CV was as high as 140.8%.

Table 3 shows the stepwise linear regression results using delta (Δ) 25(OH) D as dependent variable. In model 1, delta Δ IGF1, IGFBP3, and IGFBP5 were significantly associated (*P* < .05) (β values 0.42, 0.49, and 0.36, *P*-values 0.03, 0.03, and 0.006, respectively) with Δ 25(OH) D and significant inverse association with ΔIGFBP4 (β −0.71, *P* = .02). In model 2, only ΔIGFBP5 was significant positive association (β 0.21, *P* < .036). Similarly in models 4 and 5, Δ-IGF1 was positively associated with Δ 25(OH) D (β 0.17, *P* = .02) while Δ-IGF1/IGFBP3 was inversely associated (β −0.12, *P* = .01) with Δ 25(OH) D in the responder group. No significant relationship was found in the nonresponder group. In clinical parameters, Δ triglycerides and ΔIGF-II was inversely associated with Δ25 (OH) D.

**Table 3:**
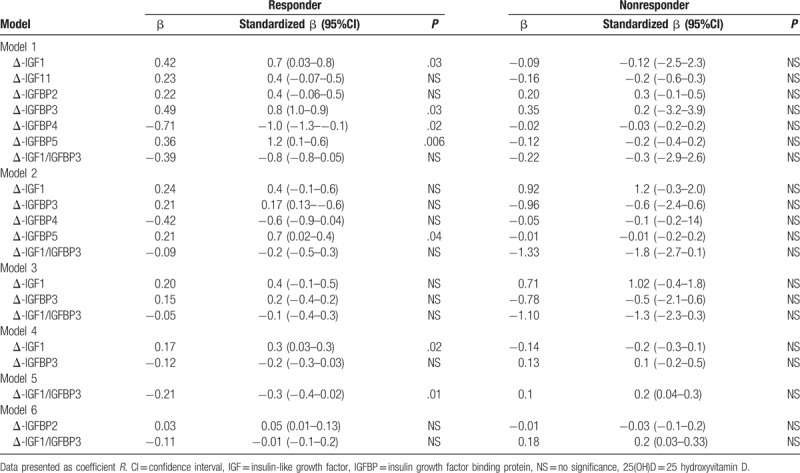
Associations of Δ 25(OH) D (dependent variable) with other parameters.

## 4 Discussion

To the best of our knowledge, this is the first study to determine IGF and IGFBP changes between responders and nonresponders to vitamin D supplementation in Saudi subjects. Nonresponders varied from responders in that they had low triglycerides and high IGF-II, IGFBP-2, and IGF-1/IGFBP-3 ratio than responders. IGF-1/IGFBP-3 ratio was considered an alternate for IGF-1 bioactivity.^[16]^ Our results, showing a significant increase in both IGF-I and IGF-1/IGFBP-3 ratio following vitamin D treatment, confirm many earlier studies in healthy subjects.^[10–14,17,18]^

In vitro studies showed that IGF-1 treatment leads to increased 1,25(OH)D2 through activating 1α-hydroxylase expression.^[11,18,19]^ Current information in human implies that this correlation is somewhat causal. Vitamin D3 supplementation significantly increases serum IGF-I and IGFBP-3 in vitamin D deficient subjects.^[14,20]^ Alternatively, short time supplementation of IGF-I raised up 1,25(OH)D2 in healthy subjects.^[12,21]^ Consequently, it can be hypothesized that vitamin D supplementation is useful in increasing IGF-I and IGFBP-3 levels. However, it should be recognized that simultaneous increase in IGF-I and IGFBP-3 concentrations may cause low or high free IGF-I available for endocrine actions.

Although there is no definite mechanism(s) through which vitamin D changes IGF-I and IGFBP-3 levels, it was confirmed that IGF-I prompt 1,25(OH)D2 synthesis in the kidney.^[9]^ Vitamin D acts in the liver and is considered the main organ for most blood IGF-I and IGFBP-3. Other related studies suggest that vitamin D stimulates production of IGF-I and IGFBP-3 in the liver.^[9]^ Furthermore, vitamin D may increase IGF-I level by increasing intestinal calcium absorption, as calcium-rich diet has been found to normalize IGF-I levels in VDR/mice^[22]^ and calcium intake was positively correlated with circulating IGF-I in humans.^[23]^

We did not find any positive correlation between 25(OH)D and IGF-1. However, an inverse association between IGF-I/IGFBP-3 ratio and 25(OH)D in the studied subjects could show that the IGF-I/IGFBP-3 molar ratio is a more sensitive marker of metabolic efficacy of IGF-I, which is online with other previous observations.^[7,24]^ Another study found a decrease in the IGF-I/ IGFBP-3 molar ratio at high vitamin D quartiles in severe obese subjects and low IGF-I/IGFBP-3 ratio following vitamin D supplementation in overweight patients.^[25]^

This study has certain limitations. First, data for vitamin D dietary intake and sun exposure were not involved and these are major factors affecting vitamin D status. Second, the results may not be generalized to other populations. Another potential limitation is that measuring IGF using new multiplex-bead immunoassay essentially affects results obtained than other immunoassays. Different assay kits for IGF-I can give varying results for the same sample, with up to a 2.5-fold difference between the lowest and highest values.^[26]^ This intermethod variability is due to calibration against different IGF-I reference preparations^[27]^ and methods used to remove IGF-binding proteins (IGFBPs).^[28]^ Interassay differences in IGF-I reference intervals are a well-known issue that has previously been underlined by many researchers.^[29–34]^ In theory, this should not be a problem in clinical practice because kits that give higher values should have higher normal limits, and patients should thus be consistently classified. This could nevertheless have important implications for diagnosis and therapeutic decision-making because a given patient could be classified as having a normal IGF-I concentration with one method but an abnormal value with another method. It is currently difficult to monitor an individual patient with different IGF-I assays, even if the results are all expressed in the same units (ng/mL). We need to establish reference intervals for multiplex-bead IGF immunoassay assay in a large background population.

## 5 Conclusion

Subjects who started with vitamin D supplementation at a lower baseline serum IGF-II, IGFBP-2, and IGF-1/IGFBP-3 ratio were more sensitive to vitamin D supplementation. Vitamin D has been shown to increase circulating IGF-I and IGFBP-3, with the consistent finding of an inverse correlation between 25(OH)D and IGF-I/IGFBP-3 in population-based cohorts of Saudi overweight subjects. Our study suggests that the modulation of circulating IGF-I and IGFBP-3 might subtend some of the beneficial health effects ascribed to vitamin D. We suggest that IGF-I and IGFBP-3 be taken into consideration in future vitamin D investigations. Furthermore, the increase in vitamin D supplementation and IGF-I/IGFBP-3 molar ratio is a more sensitive marker for the response to vitamin D supplementation in such population.

## Acknowledgments

The authors are grateful to the Deanship of Scientific Research, King Saud University for funding through Vice Deanship of Scientific Research Chairs. The authors thank PMCO who also provided technical support for the analysis of samples. The authors also thank the volunteers and the research team from the different primary care centers for the recruitment of subjects.

## Author contributions

**Conceptualization:** Nasser Al-Daghri, Spiro Garbis, George Chrousos, Majed Alokail.

**Data curation:** Kaiser Wani.

**Formal analysis:** Kaiser Wani, Malak Khattak, Spiro Garbis.

**Funding acquisition:** Nasser Al-Daghri.

**Investigation:** Kaiser Wani, Malak Khattak, George Chrousos, Omar Al-Attas, Majed Alokail.

**Methodology:** Sobhy Yakout, Spiro Garbis.

**Project administration:** Omar Al-Attas.

**Supervision:** Omar Al-Attas, Majed Alokail.

**Writing – original draft:** Sobhy Yakout.

**Writing – review & editing:** Nasser Al-Daghri, Malak Khattak, Spiro Garbis, George Chrousos, Omar Al-Attas, Majed Alokail.
